# READv2: advanced and user-friendly detection of biological relatedness in archaeogenomics

**DOI:** 10.1186/s13059-024-03350-3

**Published:** 2024-08-12

**Authors:** Erkin Alaçamlı, Thijessen Naidoo, Merve N. Güler, Ekin Sağlıcan, Şevval Aktürk, Igor Mapelli, Kıvılcım Başak Vural, Mehmet Somel, Helena Malmström, Torsten Günther

**Affiliations:** 1 Human Evolution, Department of Organismal Biology, Uppsala University, Uppsala, Sweden; 2 Ancient DNA Unit, Science for Life Laboratory, Department of Archaeology and Classical Studies, Stockholm University, Stockholm, Sweden; 3 Centre for Palaeogenetics, Stockholm, Sweden; 4 Department of Health Informatics, Graduate School of Informatics, Middle East Technical University, Ankara, Turkey; 5 Department of Biological Sciences, Middle East Technical University, Ankara, Turkey; 6 Ancient DNA Unit, Science for Life Laboratory, Department of Organismal Biology, Uppsala University, Uppsala, Sweden; 7 Present Address: Estonian Biocentre, Institute of Genomics, University of Tartu, Tartu, Estonia; 8 Department of Bioinformatics, Graduate School of Health Sciences, Hacettepe University, Ankara, Turkey

**Keywords:** Ancient DNA, Relatedness, Kinship, Archaeogenomics, Software

## Abstract

The advent of genome-wide ancient DNA analysis has revolutionized our understanding of prehistoric societies. However, studying biological relatedness in these groups requires tailored approaches due to the challenges of analyzing ancient DNA. READv2, an optimized Python3 implementation of the most widely used tool for this purpose, addresses these challenges while surpassing its predecessor in speed and accuracy. For sufficient amounts of data, it can classify up to third-degree relatedness and differentiate between the two types of first-degree relatedness, full siblings and parent-offspring. READv2 enables user-friendly, efficient, and nuanced analysis of biological relatedness, facilitating a deeper understanding of past social structures.

**Supplementary Information:**

The online version contains supplementary material available at 10.1186/s13059-024-03350-3.

## Background

The analysis of biological relatedness has become an established part of the archaeogenomic toolkit [1, 2]. It has provided us with important insights into the social structures of prehistoric groups [3–26], including Neandertals [27]. Furthermore, it serves as a quality control (QC) step in many bioinformatic pipelines to identify sample duplicates or exclude close relatives from population genomic analyses. This development has been facilitated by advances in both ancient DNA wet lab procedures and specifically designed bioinformatic methods, as the specific properties of ancient DNA do not allow the application of most approaches used with modern DNA [2]. Studies of biological relatedness in prehistoric groups are now reaching up to 100 and more individuals from the same site [22, 26], highlighting the need for further development of methods to produce optimized and efficient tools in this area.

In 2018, we published READ (Relationship Estimation from Ancient DNA) [28] as one of the first tools specifically designed to infer biological relatedness from ultra-low coverage ancient DNA data. READ uses pseudo-haploid input data and divides the genome into 1-Mbp windows, estimating the pairwise mismatch rate (P0) [29] per window and then using the genome-wide mean for relationship classification. The values are normalized by the expected P0 for an unrelated pair of individuals from the same population to account for differences in background relatedness due to population diversity and SNP ascertainment. READ then uses this normalized P0 to classify pairs of individuals as identical/twins, first-degree relatives (parent–offspring and full siblings), second-degree relatives (nephew/niece-uncle/aunt, grandparent-grandchild or half-siblings), or unrelated. This has been shown to work quite well with as little as 0.1 × shotgun coverage per genome [28]. Recent years have seen the introduction or application of more advanced methods into the field which work with lower amounts of data (Table 1), provide resolution for more distant degrees of relatedness, and/or are able to differentiate between different types of relationships for the same degree (e.g., parent–offspring versus full siblings) [30–36].

**Table 1:** Comparison of various available kinship inferring methods in terms of minimum coverage and minimum inferred relationship degree. The values shown here are retrieved from the original papers. It is important to note that the numbers of SNPs and coverages are not always straightforward to compare between studies due to differences in (the size of) the underlying SNP panel and the background relatedness of the population

Method	Min. coverage	Size of SNP panel used	Degree up to	Within-degree differentiation	Type of input
lcMLkin [30]	2 ×	100,000	Third	First-degree^g^	Biallelic genotypes or genotype likelihoods, and population allele frequencies^a^
ngsRelate [31]	1 ×	100,000	Third	First-degree, some second-degree, and some third-degree^g^	Genotype likelihoods and population allele frequencies^b^
TKGWV2 [32]	0.026 ×	~ 22,000,000	Second	None	Pseudo-haploid genotypes and population allele frequencies
KIN [33]	0.05 ×	Not specified	Third	First-degree	Aligned reads^c^ (.bam files)
BREADR [34]	0.04 ×	~ 29,000,000	Second	None	Pseudo-haploid genotypes
ancIBD [36]	0.25 × (WGS) or 1 × (1240 k SNP capture)	~ 1,200,000	Sixth	First- and some second-degree^g^	Imputed genotypes^d^
correctKin [35]	~ 85,000 overlapping markers^e^	~ 1,200,000	Fourth	None	Pseudo-haploid genotypes
READv1 [28]	0.1 ×	~ 1,200,000	Second	None	Pseudo-haploid genotypes
READv2 (this study)	0.05 × or ~ 500^f^ SNPs (first-degree) 0.1 × or ~ 2000^f^ SNPs (second-degree) 0.3 × or ~ 15,000^f^ SNPs (third-degree)	200,000	Third	First-degree	Pseudo-haploid genotypes

^a^Estimated from the input data

^b^Optional, can be estimated from the data

^c^The required input files for KIN [the number of overlapping sites, the number of pairwise differences, and the probability of runs of homozygosity (ROH) in the windows where individuals have the same long allele sequence] are created by KINgaroo, which is provided together with the KIN package, by using these.bam files

^d^Imputed genotypes are created with GLIMPSE [37] by using aligned sequence data (.bam files) as a part of the pipeline

^e^The authors of correctKin report their results using the term “overlapping markers,” which stands for the percentage of markers shared by both genotypes

^f^Absolute numbers of SNPs, not effective numbers scaled with the normalization value

^g^This classification is not automatically performed by the tool but can be achieved by comparing different statistics against each other

Nevertheless, READ continues to be a popular tool in the field partly for its user-friendliness. Since READ uses pseudo-haploid genotype calls as input, it allows the use of the same files used for other population genetic analysis without the need to generate files including sequencing read counts, calculate genotype likelihoods, or use imputation. Furthermore, READ has very simple assumptions estimating the expected pairwise mismatch rate from the data without the need for population allele frequencies, which allows using it as part of initial QC procedures or in populations (or species) for which little additional information is available.

READ [28] had been implemented as a Python 2 script, taking plain text Plink files (tped/tfam) as input. However, the last version of Python 2 was released in 2020 and some systems have already stopped supporting the language. Furthermore, READ wrote a large number of temporary files to the hard disk which were then analyzed by a separate R script called from the Python script. The output of the R script was then again read into Python and the final output was prepared. This back and forth between two scripts in different languages created ample possibilities for incompatibilities and unhandled errors. As READ continues to be used by many researchers in the archaeogenomics community, a re-implementation in Python 3 is warranted and provides the opportunity to add new features and improvements to its resource usage to be prepared for larger datasets.

Here, we re-implement the original READ [28] (READv1 hereafter) in Python 3 as READv2. The input file format has been changed from plain text Plink tped/tfam to binary Plink bed/bim/fam, requiring less space on the hard disk. All analyses are carried out within the same Python script using *NumPy* [38] and *pandas* [39] libraries, avoiding the excessive use of temporary files and the calling of a separate R script. Furthermore, by using a simulated dataset with known relationships and aDNA characteristics, we tested different window sizes which, in READv1, had been set to a default value without proper comparison. Consequently, we change the default values, obtaining a minor gain in accuracy. When the amount of data is sufficient, we further add and test new features for classifying third-degree relatives and for distinguishing between siblings and parent–offspring when a pair has been classified as first-degree relatives. Finally, we made it possible to run READv2 with diploid data without pseudo-haploidization to allow its application on the growing number of high coverage or imputed ancient genomes, a modification which increases accuracy by using more information.

## Results

### Resource demands

**Fig. 1:**
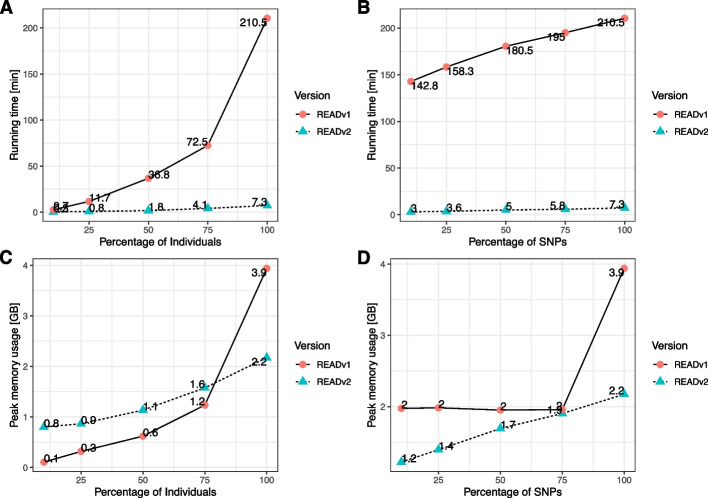
Time (**A**, **B**) and memory usage (**C**, **D**) comparison of READv1 and READv2 in a cluster node with two Intel Xeon E5 2630 v4 at 2.20 GHz/core CPUs and 128 GB RAM. The resource usage was tested with the dataset from Rivollat et al. [22] with 94 individuals. From the full data, individuals (**A**, **C**) and SNPs (**B**, **D**) were down-sampled to 75%, 50%, 25%, and 10%. Both tools were run in default settings

As some of the choices made during re-implementation are aimed at increasing the computational speed of READv2, we first test the resource demand using an empirical dataset. Rivollat et al. [22] recently reconstructed pedigrees from 94 individuals genotyped at ~ 1.15 million autosomal SNPs. READv2 analyzed the 4371 pairs in this dataset in ~ 7 min compared to 3.5 h for READv1 (Fig. 1A, B). This substantial performance gain can be attributed to the use of binary input files, loading the full data into memory, and using *NumPy* [38] and *pandas* [39] for the analysis. The gain in running time comes at the cost of an increased memory demand when the number of individuals is small (Fig. 1C), but the memory demand is lower than for READv1 for larger sample sizes and SNP numbers. The required RAM is well within the standard resources provided by current personal computers. Running time scales approximately quadratically with the number of individuals (Fig. 1A) due to the pairwise comparisons, and linearly with the number of SNPs (Fig. 1B). Notably, READv1 requires a certain amount of memory allocation (~ 2 GB) regardless of the number of SNPs (Fig. 1D), likely related to the fact that the number of 1-Mbp windows is independent of the total number of SNPs while larger numbers of SNPs require additional memory. Aside from this exception, the memory allocation follows similar trends as the running time (Fig. 1C, D). Using READv2, it was even feasible to analyze a full simulated dataset of 696 individuals and 241,860 pairs of individuals (see the “Methods” section and [40]) in less than 163 min requiring ~ 65 GB of memory. This highlights that even for such extremely large datasets, READv2 provides an option to analyze the full dataset at once if enough memory is available (e.g., on clusters).

### Window size

**Fig. 2:**
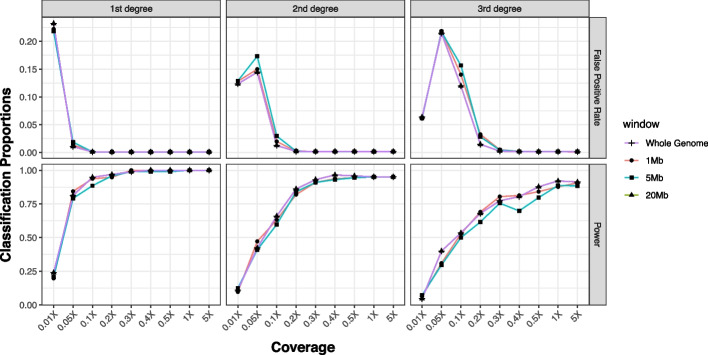
The power (i.e., the proportion of correctly classified pairs) and false positive rates (proportion of unrelated pairs classified into the respective degree) of READv2 assignment using simulated first-degree (*n* = 118), second-degree (*n* = 150), and third-degree pairs (*n* = 144). The analyses were performed using varying window sizes (1 Mb, 5 Mb, 20 Mb) (additional window sizes are shown in Additional file 1: Fig. S1) and for the genome-wide estimate (“Whole genome”), and also using varying coverages (0.01 × , 0.05 × , 0.1 × , 0.2 × , 0.3 × , 0.4 × , 0.5 × , 1 × , 5 ×). Classification proportions are shown in Additional file 1: Fig. S2. Overall, the genome-wide estimate performs better than any of the window-based methods

**Fig. 3:**
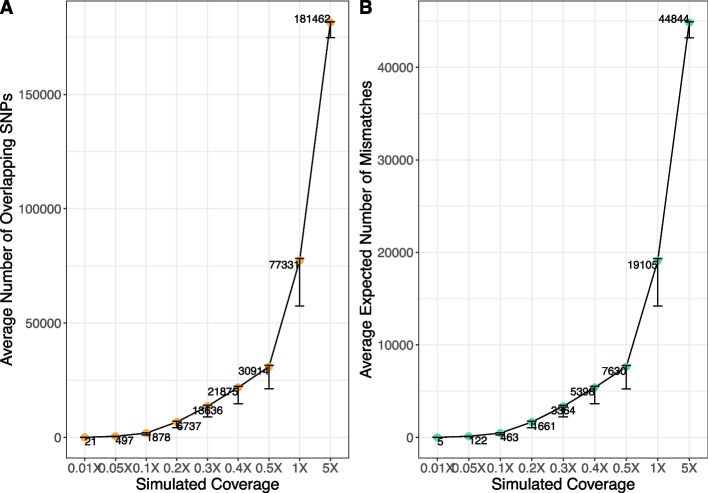
Number of overlapping SNPs in the simulated dataset (corresponding to the analysis shown in Figs. 2 and S1). Averages of the number of overlapping SNPs out of 200,000 (**A**) and the expected number of mismatches (**B**) are shown for each simulated coverage. The error bars show the minimum and maximum values of each corresponding measure. The expected number of mismatches is calculated by the multiplication of the number of overlapping SNPs and the expected pairwise mismatch proportion of unrelated individuals

READv1 [28] used a default window size of 1,000,000 bp which was inspired by GRAB [41], but it was never tested whether other window sizes could result in better accuracy. Therefore, we tested different window sizes (ranging from 100 kbp to 20 Mbp) on simulated data with known degrees of relationship [40]. In addition to this window-based approach where the test statistic P0 is estimated from the mean across all windows, we tested calculating a genome-wide P0 without splitting the genomes into separate windows. Interestingly, READv2 seemed to perform slightly better for smaller compared to larger window sizes, and overall the genome-wide estimate worked best (Fig. 2, see Additional file 1: Fig. S1 for additional window sizes). The differences are more pronounced for second-degree relationships. At 0.05 × and 0.1 × , we observe high false positive rates for second- and third-degree relatedness as many unrelated pairs are classified into these categories (Additional file 1: Fig. S2). At 0.01 × , unrelated individuals are even classified as first-degree or identical twins (Additional file 1: Fig. S2), resulting in a reduced false positive rate for second- and third-degree but an increased false positive rate for first-degree. Overall, READv2 performs well down to at least 0.1 × sequence data in the simulated dataset. This corresponded to on average about 1878 overlapping SNPs for each pair of individuals at an expected mismatch for unrelated individuals of ~ 0.247 (Fig. 3). For the implementation of READv2, we set the genome-wide estimates as default, but users can adjust the settings if they wish to use different window sizes. All analyses below are based on the new default settings.

We need to note that the normalization value, i.e., the expected pairwise mismatch between unrelated individuals, can be seen as a useful approximation for the average amount of information per SNP in a dataset. The average P0 for unrelated pairs is expected to reflect average heterozygosity under Hardy–Weinberg equilibrium for the SNP set used and it can vary substantially between populations and ascertainment schemes [28]. Therefore, an accurate assessment of the performance requires taking both the number of overlapping SNPs as well as the average mismatch for unrelated pairs into account. We use the product of these two values as the expected number of different alleles between the sample pair if they were unrelated (or “expected number of mismatches” in short) for our analyses below in order to make the number of SNPs needed more comparable across datasets with different SNP panels or population background diversities. The “expected number of mismatches” can thus be considered to represent a measure of the amount of information available for a pair to be used in kinship estimation.

### Third-degree relationships

For READv1, we did not introduce the option to classify pairs of individuals as third-degree relatives. One reason was that we expected most applications with very low coverage data, so the ranges for second-degree, third-degree, and unrelated pairs would overlap substantially, leading to false classifications. Furthermore, the 1000 Genomes Project [42] dataset that was used for testing only included a very limited number of third-degree relatives. Nevertheless, other researchers have modified READv1 to classify up to third-degree relatives [25], suggesting that the READ approach might be able to perform such classifications in certain situations. For Fig. 2, we also tested the ability of READv2 to classify third-degree relatives. As expected, the third-degree classification requires more data than second- or first-degree classifications. For low amounts of data, we see third-degree relatives frequently being assigned to other categories and unrelated pairs being classified as third-degree (Additional file 1: Fig. S2). From about 0.3 × sequencing coverage, power (~ 77% for 3rd, ~ 93% for 2nd, and ~ 98% for 1st) and false positive rates (less than 3% for all degrees) are at acceptable levels for all three categories (Fig. 2). Therefore, we decided to implement a threshold for the amount of overlapping data below which pairs falling into the third-degree category are automatically classified as “Unrelated/consistent with third degree” while a confident third-degree classification is performed for larger amounts of data. Sequencing coverage of 0.2 × corresponds to ~ 6700 overlapping SNPs in this simulated data or ~ 1700 “expected mismatches” when this value is multiplied by the expected distance of unrelated pairs. To avoid false classifications in empirical data, we implement a conservative threshold of 3000 “expected mismatches,” below which we do not attempt to classify third-degree relatives.

### Distinguishing between parent–offspring and siblings

**Fig. 4:**
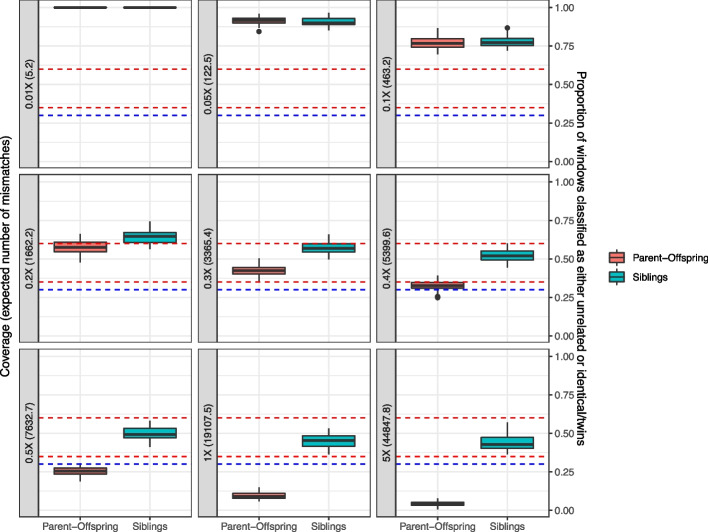
Proportion of windows that are classified as either unrelated or identical/twins. The analysis was done by using a window size of 20 Mb with 68 parent–offspring and 49 sibling pairs. Dashed lines indicate the thresholds chosen to distinguish between parent–offspring and siblings in the classification. The area under the blue dashed line shows the “parent-offspring” zone, while the area between the red lines presents the “siblings” zone. The separation is clear for coverages over 0.5 × and roughly 8000 expected mismatches. As the coverage and the number of expected mismatches reduce, the distributions begin to overlap and the proportions increase overall. Note that the average expected mismatches are slightly different from Fig. 3 as a different subsampling of the full dataset was used for the analysis in Figs. 2 and 3

Based on the estimate for a normalized P0, READ classifies pairs of individuals into degrees of relationship. For first-degree relatives, two options exist: parent–offspring and full siblings. Parent–offspring pairs share exactly one chromosome for each position of the genome while siblings should approximately share zero or two chromosomes for about one-quarter of the genome each, and one chromosome for the remaining half of the genome. By plotting the variation in the pairwise mismatch rate across the genome, some studies have resolved individual pairs of first-degree relatives [22, 43], while ancIBD [36] and KIN [33] implicitly model this as part of their Hidden Markov Models (HMM). We explored whether READv2 could use the empirical distribution across windows to distinguish between parent–offspring and full-sibling relationships. Larger windows appear more suitable for this purpose (Figs. S3 and S4), as there will be very few IBD status changes along the chromosome with a segment length of 50 cM on average (where 1 cM ~ 1 Mb in humans). In default settings, READv2 will assess the degree of relationship based on a genome-wide estimate of the pairwise mismatch rate, followed by a separate round of classification for first-degree relatives based on 20-Mb windows. As a test statistic, we use the proportion of windows classified as unrelated (i.e., no shared chromosome) or identical (i.e., both chromosomes shared), corresponding to Cotterman coefficients k0 and k2 [44], respectively. As expected, this proportion is low for parent–offspring and around 0.5 for full siblings when sufficient data are available (Fig. 4). For low amounts of data, the proportion of k0 and k2 windows first starts to increase for parent–offspring pairs and later also for siblings. While the two types are well separated ≥ 0.5 × coverage in the simulated dataset (or ~ 8000 “expected mismatches”), they overlap at ≤ 0.2 × . We used these results to set thresholds for the separation of parent–offspring from full siblings based on the proportion of windows classified as unrelated or identical. A pair of first-degree relatives is classified as parent–offspring if the proportion is below 0.3, as siblings if the proportion is between 0.35 and 0.6, and as “N/A” otherwise. Since low amounts of overlapping data result in proportions > 0.6, this allows us to avoid a classification if the amount of data is insufficient. There is, however, the risk that parent–offspring would be classified as full siblings for a specific range of overlapping data (between ~ 1600 and ~ 5000 expected mismatches).

**Fig. 5:**
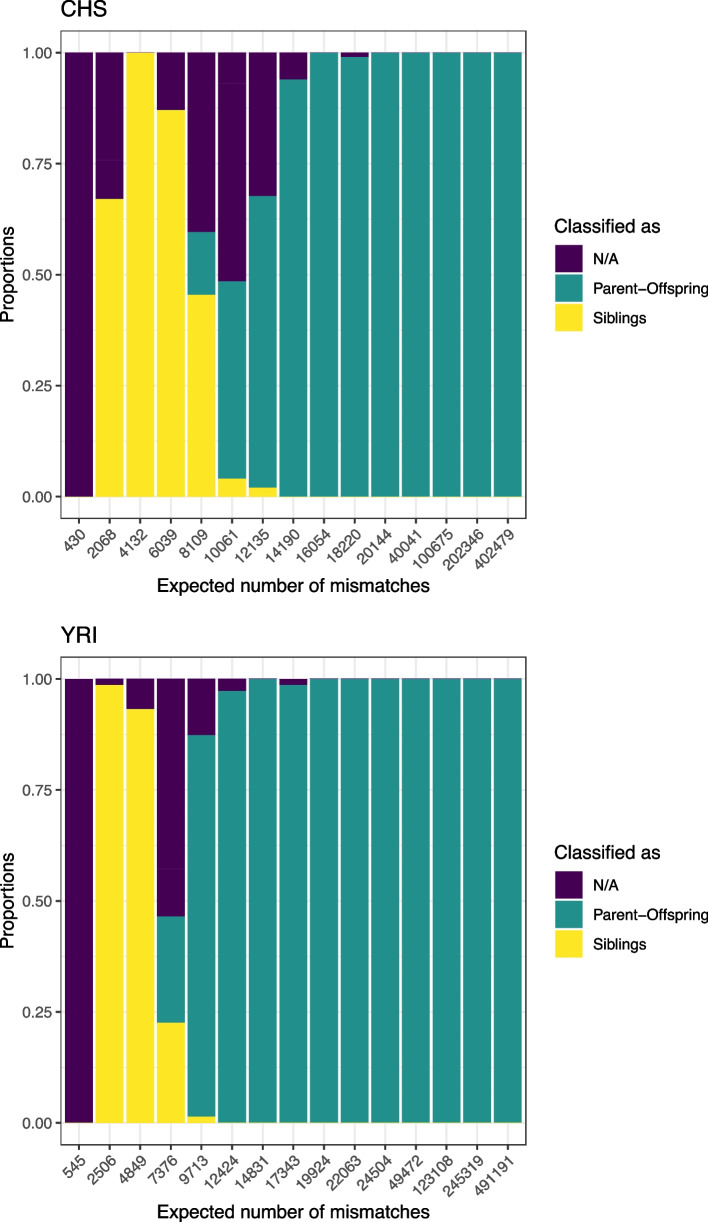
Classification of known parent–offspring pairs in empirical data. The feature was tested with CHS (Han Chinese South) and YRI (Yoruban) populations from the 1000 Genomes Project for different amounts of overlapping SNPs (*n* = 105 and 122, respectively). Similar to the result of the analysis made with the simulated data, parent–offspring pairs are correctly classified for high numbers of expected mismatches for both populations. As this number reduces, more siblings and N/A classifications start to be seen

To test this feature in an independent dataset, we selected SNP genotype data from the 1000 Genomes Project [42] where individuals from different populations have been genotyped using the Illumina Omni2.5 M chip HD genotype SNP array including 2,458,861 SNPs. We selected the populations CHS (Han Chinese South) and YRI (Yoruba) which contained the largest number of first-degree relatives: 105 parent–offspring pairs and 8 sibling pairs for CHS, and 112 parent–offspring pairs and 4 sibling pairs for YRI (Additional file 1: Table S1). The overall number of full siblings in the dataset is low, not allowing for proper testing of the feature. However, as siblings would be classified as “N/A” for increased noise, the critical test is whether parent–offspring pairs are classified as siblings at reduced amounts of data. These tests have been performed in each population separately. Similar to the simulations, parent–offspring pairs are correctly classified when the amount of overlapping data is large (> 14,000 expected mismatches, Fig. 5). Below this point, initially, parent–offspring pairs are increasingly classified as “N/A” (~ 10,000 expected mismatches). Later, we see substantial numbers of parent–offspring pairs wrongly classified as siblings. For very low amounts of overlapping data (< 2000 expected mismatches), all are classified as “N/A.” These results are qualitatively similar to the results seen for the simulated data, although the exact line where the classifications become uncertain differs slightly between datasets: ~ 8000 expected mismatches in the simulations and ~ 10,000 expected mismatches for the 1000 Genomes data. It is possible that this is related to background linkage disequilibrium which will differ between populations. By creating the founders of our simulated data from SNP allele frequency data alone, we have eliminated any existing background linkage disequilibrium in the simulated population. To be conservative and avoid wrongly classifying parent–offspring pairs as siblings, we implement a default cutoff of 10,000 expected mismatches below which classification is not performed.

### Comparison of samples with diploid genotypes

**Fig. 6:**
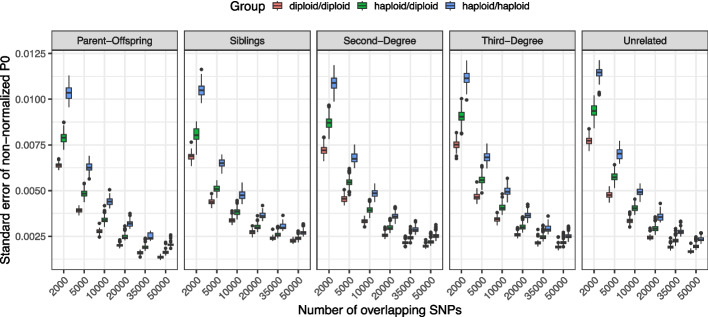
Standard errors of non-normalized P0 values calculated using diploid-diploid, haploid-diploid, and haploid-haploid comparisons based on simulated genome pairs with known relatedness degrees. Different amounts of overlapping SNPs ranging from 2000 to 50,000 are shown on the *x*-axis

Diploid ancient genomes are becoming increasingly available through high-coverage sequencing of well-preserved samples or imputation [37, 45, 46], which motivated us to modify the READ algorithm to allow processing of diploid data, in addition to pseudo-haploid data. READv2 now accepts data where pairs can have either of diploid-diploid, diploid-haploid, and haploid-haploid genotypes as input (in a diploid-haploid pair, one would have a diploid and the other a pseudo-haploid data) by counting heterozygous sites as half matches/mismatches. P0 statistics calculated in this manner should, theoretically, be more accurate than only using pseudo-haploid data. To test this, we studied the standard error (SE) of non-normalized P0 values in diploid-diploid, diploid-haploid, and haploid-haploid comparisons at various numbers of overlapping SNPs (Fig. 6). For all coverages and all relatedness types, the SE decreased systematically from haploid to diploid comparisons (Kruskal–Wallis test, *p* < 10^−17^). This effect was more prominent especially for low SNP numbers, i.e., cases where one individual is of exceptional preservation while only very low coverage pseudo-haploid data is available for the other. As a consequence of the more precise P0 estimates, one can expect better classification accuracy into different relatedness categories.

### Empirical data application: Rivollat et al. [22]

A recent study conducted the tremendous effort of obtaining genome-wide data for 94 individuals from the same site in Neolithic France, Gurgy “les Noisats,” and then reconstructing pedigrees for the individuals buried at the site [22]. The authors first ran READ [28] to estimate the degree of relatedness for each pair of individuals, followed by lcMLkin [30] to differentiate between parent–offspring and siblings among first-degree relationships. They further used BREADR [34] for individual pairs as well as imputation and ancIBD [36] for higher degrees. We have already shown that READv2 can process this dataset much faster than READv1 (Fig. 1A). By adding the new feature to differentiate parent–offspring and siblings, we are able to perform the analysis that originally needed two different tools with different input files in a single analysis that is orders of magnitude faster than the first step of the original analysis alone. About 94% of the pairs of individuals in this dataset have more than 40,000 overlapping SNPs at an expected pairwise mismatch rate for unrelated individuals of 0.245, i.e., the product for most of them is > 10,000 representing a situation where the new feature of READv2 should be applicable. All 86 first-degree pairs identified by READv1 in the original study were confirmed by READv2 (Additional file 2). For 81 of them, READv2 was able to discern parent–offspring and siblings, all in agreement with the pedigree in [22]. Four of the remaining five were not classified due to their low amounts of overlapping data (less than 7000 expected mismatches), and of these, two parent–offspring pairs would have been classified as siblings if the threshold had not been in place. Further, due to low coverage, two of these four were only classified by context in [22] rather than by a clear signal in the classification softwares. The fifth pair had sufficient data but fell between the ranges of sibling and parent–offspring used by READv2.

Notably, both READv1 and READv2 identified one additional pair of first-degree relatives (GLN207A-GLN279) that the original study did not detect with READv1. This likely reflects the stochasticity of random sequencing read sampling in the independent genotype calls as the original study had them just above the first-degree P0 classification threshold, while our results have them just below the threshold. The stochasticity of random sequencing read sampling manifests in slightly different normalization values (0.2453 for the original study, 0.2488 in our re-analysis). Using our slightly higher value would have led to a first-degree classification of the pair. Rivollat et al. had them as siblings in their pedigree based on the classification of other relatives and the lcMLkin results. READv2 also classified them as siblings. Another notable pair is GLN285A-GLN285B, which lcMLkin had as an outlier suggestive of a sibling relationship. READv2 classified them as parent-offspring. Rivollat et al. did not directly classify them as parent–offspring but excluded a sibling relationship due to the presence and absence of relationships with other individuals. Another pair, GLN288-GLN289B, was not classified by lcMLkin due to the low coverage of GLN289B. READv2 classified this pair as parent–offspring as also concluded by [22] due to the classification of other related individuals.

This re-analysis of the data from Gurgy “les Noisats” [22] illustrates that READv2 alone can lead to very similar results as the combination of READv1 and lcMLkin in the original study. Both of these latter approaches appear to miss some cases that were only resolved through context or by excluding certain types of relationships with additional data (e.g., uniparental markers, age at death). This highlights that READv2 can be used in such studies to resolve large pedigrees when combined with additional data. READv2 has the advantage that it is much faster than the combined approach and all results can be obtained by running a single tool.

## Discussion

We introduce a new version of the popular tool for inferring biological relatedness from ancient DNA data, READ. Firstly, READv2 is a Python 3 re-implementation of READv1 with substantially improved running times. Beyond speed, the implementation in a single language should also increase portability and avoid possible version conflicts. Secondly, READv2 has an updated default behavior as the pairwise mismatch rate is not derived from the mean across genomic windows but as a genome-wide estimate, leading to up to 5% improvement in classification accuracies. Finally, we added three new features: the ability to classify up to third-degree relatives, which requires at least 3000 expected mismatches; the ability to differentiate between different types of first-degree relationships, i.e., full siblings and parent-offspring, which requires at least 10,000 expected mismatches; and the possibility to perform comparisons involving diploid genotypes. The introduction of “expected number of mismatches” as a measurement of the amount of available data should also make studies and datasets more comparable. Previous benchmarking studies mostly compared the raw number of overlapping SNPs or sequencing depth without taking the information content per SNP into account (e.g., [40, 47]). Using “expected number of mismatches” will now increase the possibility of generalizing from such benchmarking results. At the same time, because the “expected number of mismatches” summarizes the allele frequency spectrum of the SNP panel in the population, these numbers will not be directly comparable to SNP numbers in approaches using the full population allele frequency information as additional input (Table 1).

The preparation and filtering of input data is crucial for any ancient DNA analysis. For example, cytosine deamination (causing C > T and G > A changes) would increase the amount of mismatches in pairwise comparisons using both transition and transversion sites. While our simulated data included cytosine deamination, the extent was identical for all individuals which should elevate the number of mismatches for comparisons equally. In practice, we recommend either using data where such damages have been repaired enzymatically or restricting the analysis to transversion polymorphisms as unequal levels of deamination might bias pairwise mismatch statistics. A READ-specific step before classification is the estimation of a normalization value expressing the expected P0 for a pair of unrelated individuals from the same population which serves as a baseline and helps to overcome potential biases arising from, e.g., general population diversity or SNP ascertainment. Obtaining a good estimate is crucial and in default settings, this value is estimated from the median of all pairwise comparisons in the dataset which usually works well for dataset sizes of 4 and higher if there is no genetic sub-structure within the data [28]. There might be situations, however, where that approach could fail, especially for small sample sizes, e.g., for a trio (mother, father, child where only one out of three comparisons is unrelated). Therefore, READ offers the possibility to manually input a normalization value which could be estimated from a different dataset or only using the pairwise mismatch between the parents in a suspected trio. One could even consider multiple DNA extractions from the same individual as technical replicates and then multiply their pairwise distance by 2 to obtain an expected value of unrelated individuals (as the expected normalized P0 for identical twins/duplicates is 0.5).

We introduced READv2 as a version with increased efficiency and compared it to READv1. Other studies have already covered comparing the general READ approach to other methods used in the field [32–34, 40, 47]. Methods such as ancIBD [36], KIN [33], and TKGWV2 [32] as well as the genotype likelihood-based lcMLkin [30] and ngsRelate [31] have specific advantages, either by providing more precise results with lower amounts of data or by being able to detect higher than second-degree relatedness confidently. In contrast to READ, they often require additional data preparation and/or information, such as read counts, estimation of genotype likelihoods, imputation, or population allele frequencies, which are often difficult to obtain for aDNA data or simply not available for certain populations. ancIBD [36] and KIN [33] were both specifically designed for ancient DNA data and their HMM approaches allow for the classification of higher degrees of relatedness as well as the differentiation between siblings and parent–offspring pairs. READv2 is very similar in its approach to BREADR [34] and TKGWV2 [32], with each tool having its own unique feature. READv2 has the functionality to separate the different first-degree relationships and to include diploid individuals, BREADR has a better quantification of uncertainty, and TKGWV2 works well with lower amounts of input data. We expect READv2 to find its own niche in this ecosystem of different methods. The combination of increased efficiency and READ’s user-friendliness with a single input file qualifies it as a QC step in data processing pipelines or as the first tool in an analysis of biological relatedness, which can be followed up with other tools to detect more fine-scale patterns or to verify results. Adding the possibility of differentiation between siblings and parent–offspring pairs when sufficient amounts of data are available provides additional value for such an initial analysis. This feature requires about 10,000 “expected mismatches,” which, assuming the popular 1240 K SNP capture panel with 1.15 million autosomal SNPs and European Neolithic populations, would correspond to about 0.2 × coverage per individual which is the case for a large proportion of all published human ancient genome-wide data [48]. As substantial amounts of generated data are also shotgun-sequenced, even lower coverages might allow for this type of analysis but the exact coverages and numbers of SNPs needed would depend on the SNP ascertainment and the population the study is focused on. Furthermore, the increased resolution of potentially classifying individuals as third-degree relatives for larger amounts of overlapping data (> 3000 expected mismatches) will improve the reconstruction of more complex pedigrees. Finally, we expect that the substantially improved running times make READ analysis feasible for future data sets, which will undoubtedly increase in sample sizes.

## Conclusions

We present READv2, an optimized Python 3 implementation of the most widely used tool for kinship classification from ancient DNA data. Through the newly implemented features and the increased computational efficiency, READv2 will enable user-friendly, efficient, and nuanced analysis of biological relatedness, facilitating a deeper understanding of past social structures.

## Methods

### READ re-implementation

READv1 [28] was written in Python2 and R, with an R script called from the Python script to carry out specific analyses. A description of the READ workflow can be found in the “Background” section. The first step of this project was to re-implement READv1 in Python 3, in order to update the script, increase efficiency and portability, and avoid possible version conflicts. The R script parts of READv1 were now implemented using the *Pandas* library [39] in Python 3. Furthermore, with the re-implementation, the input file format was changed to binary PLINK bed/bim/fam files using the *PLINKIO* library (https://github.com/mfranberg/libplinkio). In order to avoid excessive loops and improve the method’s runtime, the pairwise comparison was implemented with the *NumPy* [38] library. In addition to the window-based approach for estimating the pairwise mismatch rate (P0), a single genome-wide estimate using all covered sites was implemented. In this case, the uncertainty for the pairwise mismatch rate is estimated using a block-jackknife approach with block sizes of 5 Mb as commonly employed in human population genomic studies [49].

For READv1, the classification thresholds were set to the mid-point between the expected P0 values for each degree. Consequently, we also set the cutoff for third-degree classifications halfway between the expected P0 for unrelated individuals (i.e., 1.0) and third-degree relatives (0.9375). Pairs of individuals with a normalized P0 between 0.90625 and 0.953125 are now classified as third-degree relatives if the number of expected mismatches (number of overlapping SNPs times the pairwise mismatch rate expected for unrelated individuals) is 3000 or higher; otherwise, they are classified as unrelated.

To differentiate between parent–offspring and sibling pairs, the genome is divided into windows of 20 Mb and the classification is made based on the proportion of windows that are classified as either “identical/twin,” “unrelated,” or “third degree.” If that proportion is less than 0.3, the pair is classified as “parent-offspring”; if it is between 0.35 and 0.6, the pair is classified as “siblings.” For other proportions, or when the number of expected mismatches is below 10,000, the type is not specified beyond “first-degree.”

To allow for diploid-diploid and diploid-haploid comparisons, READv2 treats any comparison involving a heterozygous position in one individual as 1/2 match and 1/2 mismatch, irrespective of the other individual’s genotype. We explain the logic here using an example locus with two alleles, *A* and *a*, which presents three possibilities:

- Individual 1 has *Aa* and individual 2 has *Aa* genotype, which means 2 mismatches in 4 comparisons, and hence an average of 1/2 mismatch.
- Individual 1 has Aa and individual 2 has AA or aa genotypes, which means 2 mismatches in 4 comparisons, and hence an average of 1/2 mismatch.
- Individual 1 has Aa and individual 2 has A/a pseudo-haploid genotype, which means 1 mismatch in 2 comparisons, and hence 1/2 mismatch.

**Fig. 7:**
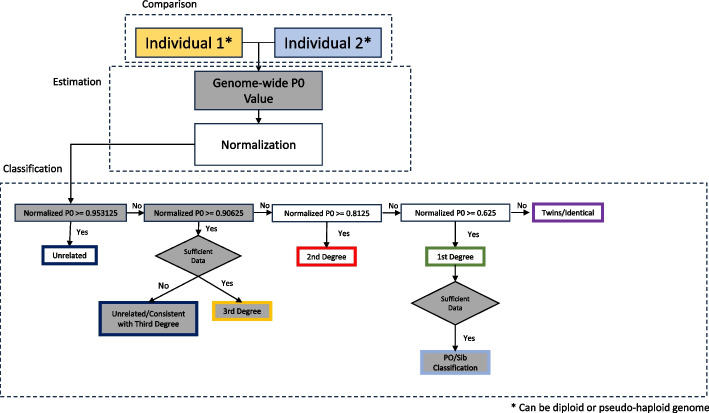
Flowchart of READv2. The novel steps and classification results that differ from READv1 have been highlighted in gray

Furthermore, we expanded the output table by including information such as the number of overlapping SNPs, the number of expected mismatches, and the kinship coefficient θ (= 1 − normalized P0) as well as its confidence interval to fulfill several user requests. An overview of the READv2 workflow can be found in Fig. 7. READv2 is available with instructions on its usage at https://github.com/GuntherLab/READv2.

### Simulated data and processing

The next step after the re-implementation was to perform a benchmark with simulated data with known relationships. We used previously simulated pedigree data comprised of first-, second-, and third-degree related ancient genomes from Aktürk et al. [40]. This pedigree simulation was performed using the pedigree simulator software PED-SIM (v 1.3) [50]. For this simulation, founder genotype data was created from scratch using 8,677,101 biallelic autosomal SNPs with MAF ≥ 0.01 among Tuscan (TSI) individuals of the 1000 Genomes Project v3 [51]. The founders were generated by choosing two alleles for each SNP proportional to their population allele frequencies. This way, 120 unrelated founders for first-degree and 240 unrelated founders for second- and third-degree pedigree simulations were generated. The method of generating founder data employed here leads to the elimination of any background relatedness among founders and the homozygosity blocks within founder genomes.

Seventy-two pedigrees for first-degree relationships, 96 for second-degree relationships, and 96 for third-degree relationships were simulated. The founders of each pedigree and simulated individuals from distinct pedigrees were treated as “unrelated.” For each relationship type, we chose *n* = 48 pairs. For instance, we simulated *n* = 72 individuals (*n* = 24 trios) for parent–offspring relationships, resulting in 48 unique pairs. Consequently, the dataset comprises 696 individuals (*n* = 72 for parent-offspring, grandparent-grandchild, and great-grandparent-great-grandchild and *n* = 96 for siblings, half-sibling, avuncular, first cousin, and grand avuncular relationships).

To test diploid-diploid and diploid-haploid comparisons, we used these simulated genotypes without missing data or ancient DNA damages. From the simulated genotypes, we chose 2000, 5000, 10,000, 20,000, 35,000, and 50,000 shared SNPs as perfect genotypes for all relatedness degrees to resemble diploid data and their pseudo-haploidized versions for comparisons. This approach allowed us to test the effect of diploid comparisons on the standard errors of P0 without being dependent on mapping, diploid calling, and imputation pipelines.

For all other testing, the simulated data was processed further to resemble realistic ancient DNA data. Out of the ~ 8.7 million SNPs, 200,000 were chosen at random for the kinship analysis of simulated ancient DNA data. Sequencing data was then generated using the *gargammel* software [52], which introduces post-mortem damage and sequencing errors to the sequencing reads. The extent of deamination damage was identical for all individuals [40]. For the processing of simulated NGS data, we followed the same approach as applied to ancient genome sequencing data in the field [14, 53]. BAM files are available from Zenodo [40, 54, 55].

Genotypes were called from the BAM files using ANGSD v0.933 [56] with the options -checkBamHeaders 0 -doHaploCall 1 -doCounts 1 -doGeno -4 -doPost 2 -doPlink 2 -minMapQ 30 -minQ 30 -doMajorMinor 1 -GL 1 -domaf 1. Pseudo-haploid tped/tfam files were then generated with the ANGSD tool haploToPlink and converted to binary Plink files using Plink [57].

### Benchmarking

In order to reduce the memory and runtime of the window size comparisons, the dataset was divided into groups of 70 by involving all related individuals, i.e., all 3 individuals (two parents and one offspring) in a parent–offspring relationship, in a group with Plink –keep-fam command. The normalization value was calculated as the median mismatch per subsample of the data. To test the performance of READ for different coverages, the original simulation data was down-sampled with SAMTOOLS view -s [58]. In order to see how window size affects the results and compare the window-based and genome-wide approaches, the power of the method (TP/(TP + FN)) and the proportion of unrelated pairs classified as related (false positive rate) were calculated for each coverage and window size (results shown in Fig. 1).

While the window size comparisons were performed on the dataset separated into groups of 70, the tests for distinguishing between siblings and parent–offspring were conducted on the full dataset at once.

Empirical data from the 1000 Genomes Project [42] with known relationships were used for further testing. The autosomal Illumina Omni2.5 M chip HD genotype SNP array data consists of 2368 individuals from 15 different populations with 2,458,861 SNPs. The populations with the most parent–offspring pairs, namely YRI (Yoruba in Ibadan, Nigeria) and CHS (Southern Han Chinese, China), were selected for further steps. The populations were separated into different.bed files with the PLINK –keep-fam option and later SNPs were down-sampled with the PLINK –thin option. Since the data was from modern samples and contained diploid genotype calls, the data were made homozygous by randomly selecting one allele at each position.

### Empirical data application

We downloaded 1240K SNP capture BAM files for 94 individuals excavated in Gurgy “les Noisats” [22, 59] from the European Nucleotide Archive [60]. Genotypes at ~ 1.15 million autosomal SNPs were called using ANGSD v0.933 [56] with the options -checkBamHeaders 0 -doHaploCall 1 -doCounts 1 -doGeno -4 -doPost 2 -doPlink 2 -minMapQ 30 -minQ 30 -doMajorMinor 1 -GL 1 -domaf 1. Pseudo-haploid tped/tfam files were then generated with the ANGSD tool haploToPlink which were converted to bed/bim/fam with Plink v1.90b4.9 [61]. We then ran READv2 in default settings. To compare the resources needed from running READv1 and READv2, we also sub-sampled the number of individuals (with the Plink command –thin-indiv) and the number of SNPs (with the Plink command –thin).

### Supplementary Information

Additional file 1: Fig. S1. The power and false positive rates of READv2 for first-degree, second-degree, and third-degree pairs with additional window sizes. Fig. S2. Proportions of simulated individuals with known biological relatedness classified into the different categories. Fig. S3. Variance of normalized P0 values along the windows of varying sizes for 1X coverage. Fig. S4. Examples of histograms of normalized P0 values for simulated parent-offspringand siblingpairs for varying window sizes. Table S1. The number of Parent–Offspring and Sibling pairs present in the genotyping data in populations from the 1000 Genomes Project

Additional file 2. READv2 results for the Rivollat data [22]. Explanations of the columns can be found in the second tab. This table also represents an example of the output of READv2.

Additional file 3. Review history

## Data Availability

An open-source application of READv2 is available under a GPL-3.0 license on GitHub [62] and bioconda [63]. We have deposited the version of the software used in the manuscript on Zenodo [64]. Simulated data used for benchmarking is available from Zenodo [54, 55]. Genotype data from the 1000 Genomes Project is available at [65]. Empirical ancient DNA NGS data was previously published and is available at the European Nucleotide Archive [66].
