# Supplementary material for: READv2: advanced and user-friendly detection of biological relatedness in archaeogenomics
Source: Genome Biol. 2024 Aug 12;25:216. doi: 10.1186/s13059-024-03350-3 (PMC11318251; doi:10.1186/s13059-024-03350-3)
Supplement: Supplementary file 1 — Additional file 1: Fig. S1. The power and false positive rates of READv2 for first-degree, second-degree, and third-degree pairs with additional window sizes. Fig. S2. Proportions of simulated individuals with known biological relatedness classified into the different categories. Fig. S3. Variance of normalized P0 values along the windows of varying sizes for 1X coverage. Fig. S4. Examples of histograms of normalized P0 values for simulated parent-offspringand siblingpairs for varying window sizes. Table S1. The number of Parent–Offspring and Sibling pairs present in the genotyping data in populations from the 1000 Genomes Project [file 13059_2024_3350_MOESM1_ESM.pdf]

# Additional File 1

## READv2: advanced and user-friendly detection of biological relatedness in archaeogenomics

Erkin Alaçamlı, Thijessen Naidoo, Merve N. Güler, Ekin Sağlıcan, Şevval Aktürk, Igor Mapelli, Kıvılcım Başak Vural, Mehmet Somel, Helena Malmström, Torsten Günther

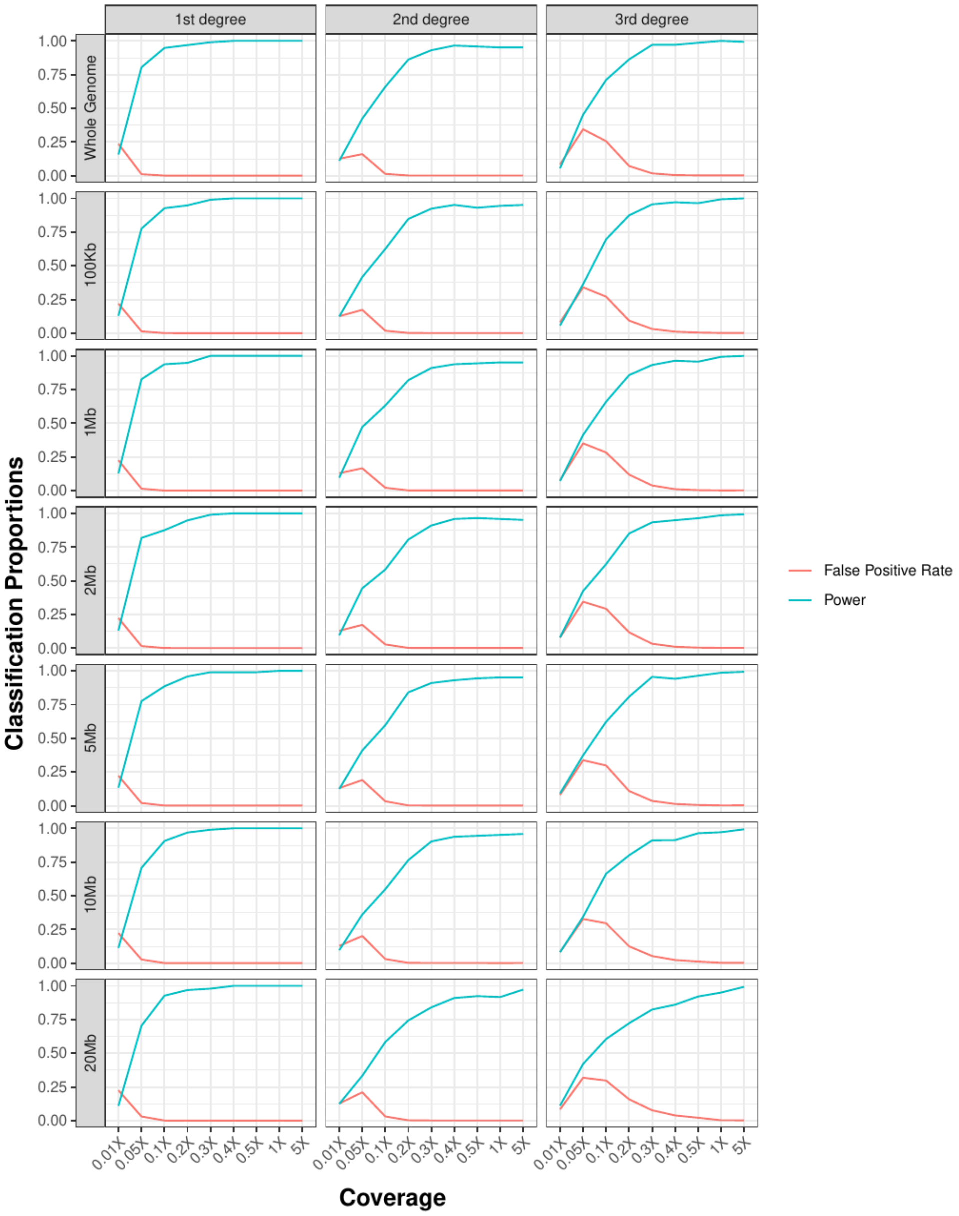


**Figure S1**: The power and false positive rates of READv2 for first-degree, second-degree, and third-degree pairs with additional window sizes. As also shown in Figure 2, READ performs well for coverages over 0.1X to classify first-degree pairs and over 0.3X for second- and third-degree pairs. Although there is not much difference between window sizes, the genome-wide estimate works the best overall.

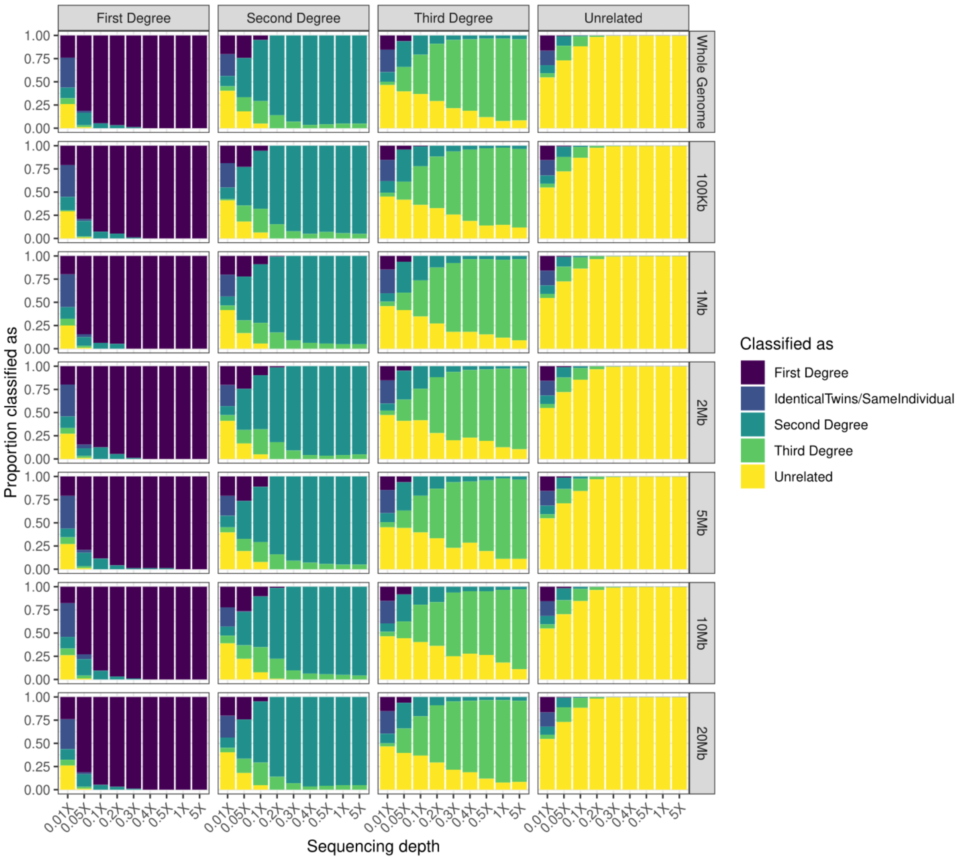


**Figure S2**: Proportions of simulated individuals with known biological relatedness classified into the different categories. Columns correspond to the true relationship while rows show the different window sizes, and colors represent the classification outcomes.

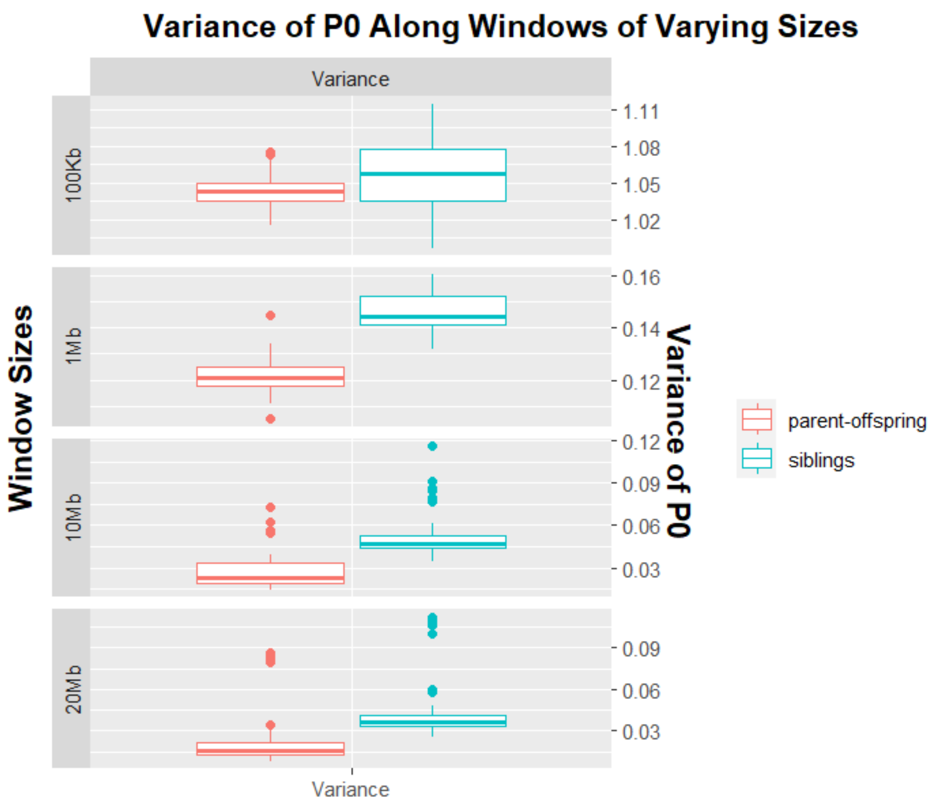


**Figure S3**: Variance of normalized P0 values along the windows of varying sizes for 1X coverage. The variance of parent-offspring and sibling pairs are visibly separated for large window sizes (1Mb, 10Mb, and 20Mb). However, as the window size decreases, that clear separation is lost. Moreover, the scale differs between window sizes used.

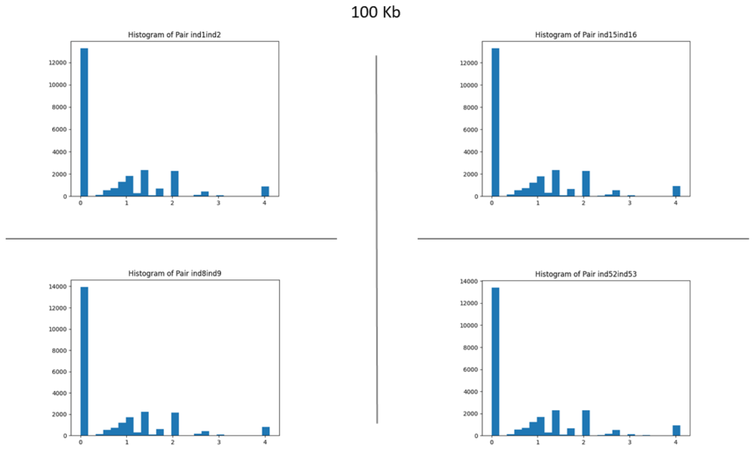

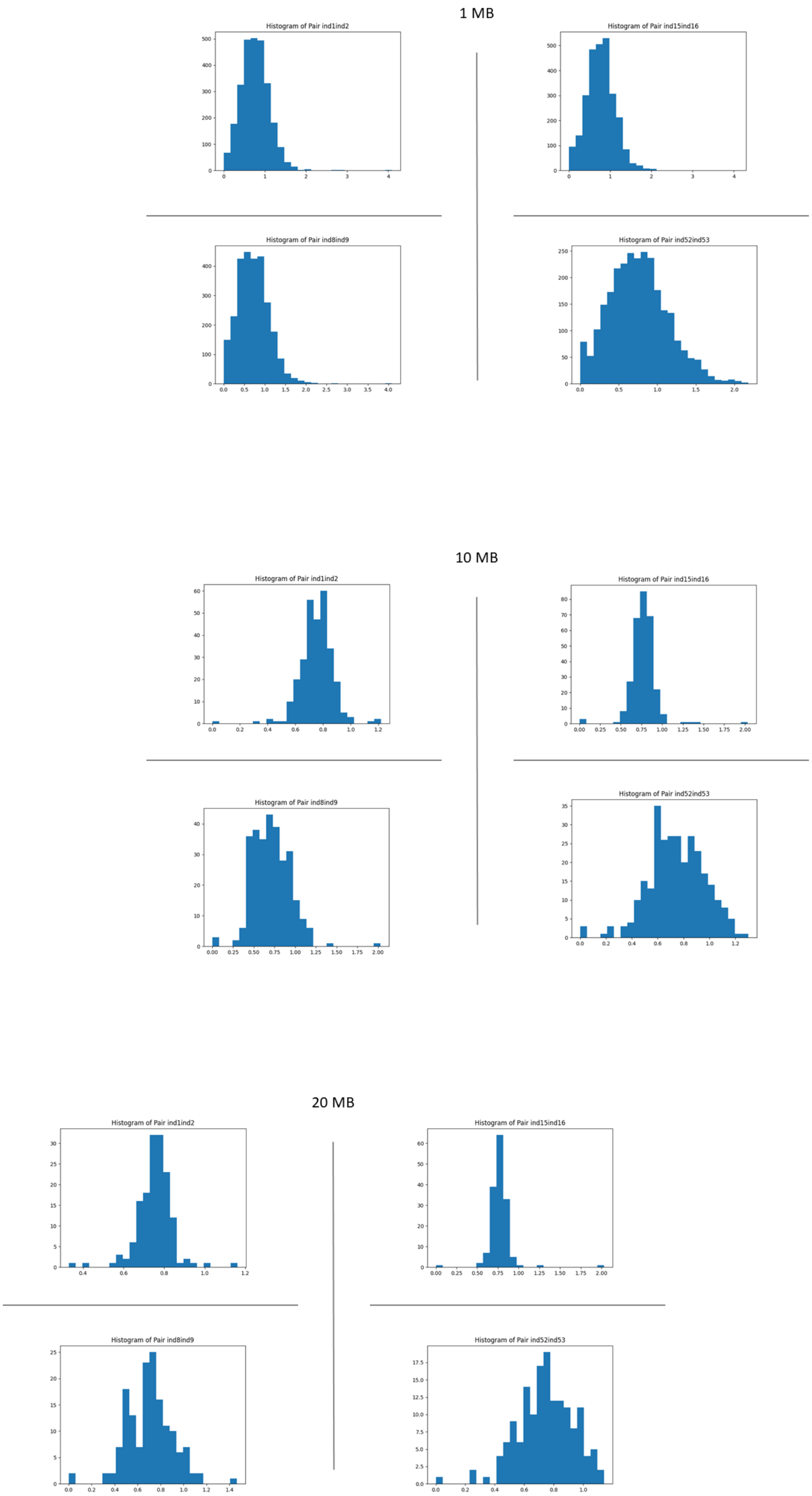


**Figure S4**: Examples of histograms of normalized P0 values for simulated parent-offspring (top) and sibling (bottom) pairs for varying window sizes. Smaller window sizes show very noisy distributions for parent-offspring and sibling pairs. However, windows of 20Mb result in distributions centered in the first-degree region with wider distribution for sibling pairs.

**Table S1**: The number of Parent-Offspring and Sibling pairs present in the genotyping data in populations from the 1000 Genomes Project.

| Population Name | Number of Parent-Offspring Pairs | Number of Sib. Pairs |
|---|---|---|
| ASW | 46 | 8 |
| CDX | 2 | NA |
| CEU | 1 | NA |
| CHS | 105 | 8 |
| CLM | 69 | NA |
| GBR | 2 | 1 |
| GIH | NA | NA |
| IBS | 100 | NA |
| KHV | 41 | 1 |
| LWK | 5 | NA |
| MXL | 60 | 3 |
| PEL | 70 | 1 |
| PUR | 66 | NA |
| TSI | 1 | NA |
| YRI | 112 | 4 |
