# Supplementary material for: READv2: advanced and user-friendly detection of biological relatedness in archaeogenomics
Source: Genome Biol. 2024 Aug 12;25:216. doi: 10.1186/s13059-024-03350-3 (PMC11318251; doi:10.1186/s13059-024-03350-3)
Supplement: Supplementary file 3 — Additional file 3. Review history [file 13059_2024_3350_MOESM3_ESM.docx]

**1^st^ round**

**Reviewer 1**

In this manuscript, the authors introduce a computational method to calculate pairwise biological relatedness in ancient DNA samples ("READ_V2"). The software is an update of a popular tool widely used in human aDNA analysis ("READ"). Both versions implement a simple but robust approach of using the pairwise allelic mismatch rates between two samples to infer close biological relatives.

READ_V2 is a re-implementation purely in Python code, adding a slight update to the default calculation settings (leading to a minor improvement), much-improved runtimes, and two new functions:

A) Inferring 3rd-degree relatives (if enough data is available) and

B) Distinguishing between parent-offspring and full siblings - as only the latter have varying biological relatedness along the genome.

I believe that these updates make a substantial improvement and that the aDNA community will widely use "READ_V2". Several similar methods now implement the simple (but highly robust and practical) signal of average pairwise mismatch rates, where READ_V2 stands out for its ease of use and sensible default choices.

The tool is available as a Python file, and I could run it right away on the example data the authors provide (less than 5 minutes from download to the inference results).

Overall, I find the article accessible, well written, and easy to follow.

Below, I list major comments (A), minor comments (B), and potential suggestions to increase adoption (C).

I stress that none of those points are make-or-break, as the presented data backs up all the claims in the manuscript, and the software READ_V2 is ready for use. However, I hope the authors find some of my comments helpful in improving the presentation and reaching an even wider community with their excellent tool.

Harald Ringbauer

##############

A Major Comments:

A1)

L104-106: "Subsampling to 50% of the individuals reduced the running time of READv2 to about one quarter (Figure 1A), suggesting a quadratic relationship due to the pairwise comparisons made"

In Fig. 1, you only show two points (half SNPs and half IIDs), which de facto does not distinguish between exponential/quadratic/linear scaling of runtime and memory usage. It would be very helpful to vary the number of test points (#SNPs and #IIDs) over multiple values and show the runtime and memory as curves.

Runtime and memory scaling is a key metric of computational software, and there are some simple intuitive behaviors (e.g., quadratic scaling with number IIDs, as the number of pairs between n samples grows as n(n-1)/2; or linear memory requirement with #SNPs or #individuals) that one would ideally confirm.

A2)

L151: "the number of overlapping SNPs as well as the average mismatch for unrelated pairs into account. We use the product

of these two values as the "effective number of overlapping SNPs"

I find the term "effective number of overlapping SNPs" to be a bit misleading. As can be directly seen, the value is the *expected number of different alleles between this sample pair if they were unrelated*. So something like "expected mismatches" would be more fitting.

I suggest explicitly describing this helpful interpretation.

A3) In Figure 2 and Figure 3, you show odd and non-matching numbers of simulation replicates. E.g., in Fig. 2 caption: "using simulated first-degree (n=118), second-degree (n=150), and third-degree pairs (n=144)".

It would be easier to describe and understand if a matching number is shown (and perhaps a larger number such as n=500, as n=100 can have substantial sampling uncertainty for intermediate fractions).

A3) L381-L383:

"READv2 is very similar in its approach to BREADR [33]

and TKGWV2 [31], with each tool having its own unique feature. READv2 has the

functionality to separate the different first-degree relationships, BREADR has a better

quantification of uncertainty, and TKGWV2 works well with lower amounts of input data."

You summarize the relationship to other methods that use the same signal to detect relatives in two brief sentences. I think it would help to expand this discussion of the pairwise mismatch estimate ecosystem, and where you think READv2 will find its niche.

A4) L473: "The normalization value was calculated as the median mismatch per subsample

of the data."

This sentence in the section "Benchmarking" is the only time normalization and using the median are mentioned in the manuscript. Normalization is a key choice, and the online documentation discusses it extensively. It could help to move some of these explanations into the manuscript.

B) Minor suggestions:

B1)

L108: "Using READv2 it was even feasible to analyze a full simulated dataset of 696 individuals and

241,860 pairs of individuals (see Methods and [38]) in less than 4 hours, but this required

237.5 GB of memory."

As a reader, one immediately compares these values to the "94 IIDs" and "5.64 GB" you mentioned above in the manuscript. My question was why the memory requirement is so much larger than just the factor of 7x (due to 7x more individuals). I guess that is the number of SNPs in the two datasets (1.2 million 1240k SNPs versus 8.7 million simulated WGS SNP), but it would be less of a "jump scare" if you make this point explicitly.

B2)

L131: "READv2 seemed to perform slightly better for smaller compared to larger window sizes, but overall the genome-wide estimate worked best".

Isn't the genome-wide estimate effectively window size 1? So there is no "but".

B2)

L363 I had to look up the Latin abbreviation "viz.". Perhaps write "namely," as the Latin abbreviation seems to decline in use (even some native speakers do not know it anymore).

B3) In Table 1, only integers of the "Average number of effectively overlapping SNPs" are shown - which gets very coarse for low numbers (e.g., 5 in the last row). For low values, it would be useful to show at least two significant digits.

Overall, that table could also easily be shown as a figure - where one would see the "scaling" with coverage more accurately and at one glance.

B4) L216: "Larger windows appear more suitable for this purpose (Figures S3 and S4), so we perform this analysis with 20Mb windows."

You do not give any actual reason why it is more useful. But the heuristic is simple—each meiosis in one of the two parents changes the IBD0/IBD1/IBD2 status, and there are only a few per chromosome. Thus, the stretches of the same status will be, on average, 50 cm long (and roughly 1cm = 1mb).

B5) L222: "Cotterman coefficients k0 and k2". I believe that a citation would help. "Cotterman" is not a widely used nomenclature\

B6) "This feature requires about 10,000 "effectively overlapping SNPs", which, assuming the

popular 1240K SNP capture panel with 1.15 million autosomal SNPs and European Neolithic

populations, would correspond to about 0.2X coverage per individual which is the case for a

large proportion of all published human ancient genome-wide data [44]"

As a substantial fraction of published aDNA is shotgun-sequenced, it would be very useful to also provide the corresponding coverage for that data type.

B7) Table S1 (the comparison of different methods to detect relatedness in aDNA) is very informative. I would suggest considering moving it into the main, as it really helps to put READv2 into a broader context of methods.

C) Potential Updates to increase User-Friendliness

Here I provide some suggestions to increase the usability further. Those are entirely optional, and the authors should only go there if they believe it is a valuable use of their time.

C1) Packaging as proper Python package on pip

The authors could make their software (or effectively the .py file) a pip installable package. While, of course, not completely trivial, it is pretty straightforward (see https://packaging.python.org/en/latest/tutorials/packaging-projects/). Having READv2 as its pip package allows proper versioning and software dependencies on the necessary Python tools. Proper packaging would be an important step to include it in a widely used aDNA pipeline (e.g., Eager, which would benefit massively from a default step of detecting relatives) - further boosting the adoption.

C2) Using eigenstrat format as input

Eigenstrat (binary and non-binary) is widely used in aDNA (e.g., the Allen Ancient DNA resource is published in eigenstrat format), and most pseudo-haploid data is in this format. Of course, it is relatively straightforward to transform eigenstrat files into the READv2 Plink files (as the authors describe in their README). However, doing so creates an extra step requiring external software. There, it would be very user-friendly to provide the ability to analyze Eigenstrat files directly.

Existing Python scripts can read both binary and non-binary eigenstat files, such as the one used by hapROH (see code at https://github.com/hringbauer/hapROH/blob/master/package/hapsburg/PackagesSupport/loadEigenstrat/loadEigenstrat.py).

C3) READv2 has the nice feature of automatically classifying relatives (or refraining from doing so) using sensible coverage and statistical cutoffs. However, it would be very useful for the user to output the uncertainty of the final test statistic (the kinship coefficient theta) used for classification. As you have the bootstrap confidence intervals for PO - you could easily forward those CIs to theta and report those in the tabular output.

**Reviewer 2**

Alaçamlı and colleagues present an exciting update to the most widely used relatedness estimator in the field of archaeogenomics. This comes several years after the first release of READ (v1), and brings a welcomed update in speed, resource usage, and importantly, accuracy. As a frequent user of READv1, I am happy to see that the researchers remain interested in their own software, to the point that new features were developed, tested, and incorporated, and that the community's comments on how to possibly improve the software were listened to.

Overall, the paper is well structured, in similar ways to other recent ancient DNA relatedness estimator papers. As the updated method is based on a peer-reviewed and well-established existing method, the authors dive right into the new features of READv2, and end up with a section where the method is applied to archaeogenomic data from a recent study.

Although I would happily recommend this paper to publication in Genome Biology, almost as is, I have a few small comments and observations that the authors could shed some more light into:

Results:

- Regarding the "Window size" section, perhaps a couple of extra sentences could be provided about the simulated data being used for these tests. Perhaps that will explain why only 1,878 SNPs overlap on average on pairs of individuals with 0.1X coverage data. From personal experience that coverage level should be able to provide way more overlapping SNPs than what is reported.

- I'm not completely convinced that using "effective number of overlapping SNPs" as a comparative threshold measure benefits the discussion and/or makes it clearer and more comparable. However, I am happy to change my mind!

Mainly because minimum SNP (and/or coverage) thresholds are often used before using these methods to decide which samples should be used for relatedness estimation. Shall users then mostly use whole-genome coverages as the selecting threshold before running READv2? What about targeted-capture coverages? Also, see my comment below about "effective number of overlapping SNPs" not being comparable to all relatedness methods.

- "Third-degree relationship": it would be useful for the reader to see false positive ratio numbers, for example when the 0.3X coverage is mentioned, just to have a more immediate understanding of the limits of this threshold.

- "Distinguishing between parent-offspring and siblings": On line 225 and after it is mentioned that from ~8000 effectively overlapping SNPs there is perfect separation between first degree classes. However, on line 262 and below (and Fig. 4) it is mentioned that 8000 effectively overlapping SNPs are still prone to misclassifications, while mentioning that the results are similar to what was achieved in the prior simulations. Are these differences due to different median/average mismatch rates? If effectively overlapping SNPs are supposed to normalise and make the results more comparable "across datasets with different SNP panels or population background diversities", how are the 8000 vs >14000 SNPs comparable to achieve similar successful classifications?

- "Empirical data application: Rivollat et al. (2023)": In lines 332-335, did the authors compare the average/median mismatch rate of the original publication and their own? Could that be also a reason why the pair "GLN207A-GLN279" cross the threshold in one run but not another?

Discussion:

- "The introduction of "effectively overlapping SNPs" as a measurement of the amount of available data should also make studies and datasets more comparable and increase the possibility of generalizing from benchmarking results as previous studies mostly compared the raw number of overlapping SNPs or sequencing depth without taking the information content per SNP into account."

This can be true for comparing relatedness methods relying on pairwise mismatch rates, but won't be comparable to the methods described in Table S1 that use allele frequencies. That should be specifically mentioned.

-Figure S1/S2: It's not crucial, but it would be interesting to also see the power and minimum data threshold of READv2 for "IdenticalTwins/SameIndividual" relationships.

-Table S1: This is an extremely useful table for the community, but where does the 200,000 SNP panel of READv2 come from? I couldn't find a reference to it in the text, as it either mentions several million SNP datasets or the 1240K capture set. Also, for third degree the ~15,000 SNP number seems to not be the effectively overlapping SNPs, which makes sense when comparing with other methods, but in my view further makes things a bit more confusing.

Finally, the fact half of my comments/observations turned out to be regarding the way SNP thresholds are presented, attests to the quality and importance of the improvements of READv2 over its predecessor, and both as a reviewer and community member I am extremely happy to see it being published. Thanks for keeping on improving software in such a quickly moving field!

**Reviewer 3**

The study presents an advancement of the widely used tool READv1, designed for estimating kinships among ancient individuals using low-coverage data. This updated tool, named READv2, has been specifically tailored for Python 3 environments, introducing enhanced functionalities. It offers additional capabilities to identify third-degree relatives, as well as distinguishing between siblings and parent-offspring, both classified as first-degree relatives. As a user of READv1, I would like to see the paper ultimately published in this journal.

I only have a couple of questions. My small concern is the robustness of READv2 against ancient DNA damages, given their potential to influence pairwise mismatch rate estimations. Would the tool's performance improve if the sites in shotgun data were filtered for transversions?

This could be tested in the context of capture-sequenced data for 1240K sites or whole-genome shotgun sequenced data with an additional filter for minor allele frequency 1% based on global populations within the 1000 Genome Phase 3 dataset.

Moreover, in instances where ancient DNA is not treated with UDG, are there any potential pitfalls in running READv2 or interpreting its results? Clarifying these aspects would greatly enhance the utility and reliability of the tool in diverse genomic contexts.

**Authors’ response**

In addition to the suggestions received from the reviewers, we have incorporated feedback received on the preprint version of the manuscript. Most importantly, we have been informed that we had made a mistake in our calculation of the classification threshold for 3rd degree relatives. We have updated the value in the code and repeated all analysis. As a consequence, we can even classify 3rd degree relatives with less SNPs at a higher accuracy. All relevant parts of the manuscript have been updated.

Finally, we have included an additional feature into the READv2 code. Previously, we assumed that all individuals are pseudohaploid. However, in certain situations, diploid calls will be available for some individuals – either due to exceptional preservation or imputation. We add the option for diploid-diploid and diploid-haploid comparison and show that such comparisons result in a more precise estimation of our test statistic P0. Please find a point-by-point response to the reviewer comments below.

Reviewer #1: In this manuscript, the authors introduce a computational method to calculate pairwise biological relatedness in ancient DNA samples ("READ_V2"). The software is an update of a popular tool widely used in human aDNA analysis ("READ"). Both versions implement a simple but robust approach of using the pairwise allelic mismatch rates between two samples to infer close biological relatives.

READ_V2 is a re-implementation purely in Python code, adding a slight update to the default calculation settings (leading to a minor improvement), much-improved runtimes, and two new functions:

A) Inferring 3rd-degree relatives (if enough data is available) and

B) Distinguishing between parent-offspring and full siblings - as only the latter have varying biological relatedness along the genome.

I believe that these updates make a substantial improvement and that the aDNA community will widely use "READ_V2". Several similar methods now implement the simple (but highly robust and practical) signal of average pairwise mismatch rates, where READ_V2 stands out for its ease of use and sensible default choices.

The tool is available as a Python file, and I could run it right away on the example data the authors provide (less than 5 minutes from download to the inference results). Overall, I find the article accessible, well written, and easy to follow.

Below, I list major comments (A), minor comments (B), and potential suggestions to increase adoption (C).

I stress that none of those points are make-or-break, as the presented data backs up all the claims in the manuscript, and the software READ_V2 is ready for use. However, I hope the authors find some of my comments helpful in improving the presentation and reaching an even wider community with their excellent tool.

Response: Thank you! We are very pleased to receive this positive assessment and we appreciate the constructive suggestions.

Harald Ringbauer

A Major Comments:

A1)

L104-106: "Subsampling to 50% of the individuals reduced the running time of READv2 to about one quarter (Figure 1A), suggesting a quadratic relationship due to the pairwise comparisons made"

In Fig. 1, you only show two points (half SNPs and half IIDs), which de facto does not distinguish between exponential/quadratic/linear scaling of runtime and memory usage. It would be very helpful to vary the number of test points (#SNPs and #IIDs) over multiple values and show the runtime and memory as curves.

Runtime and memory scaling is a key metric of computational software, and there are some simple intuitive behaviors (e.g., quadratic scaling with number IIDs, as the number of pairs between n samples grows as n(n-1)/2; or linear memory requirement with #SNPs or #individuals) that one would ideally confirm.

Response: The reviewer is correct that we cannot conclude scaling from one level of subsampling. In addition to down-sampling to 50%, we have now added 10, 25 and 75% of the total SNPs/individuals and show these different levels in Figure 1. Not all relationships seem clearly quadratic or linear, likely as certain data structures will always require a certain size (e.g. the number of windows across the genome is independent of the number of SNPs).

A2)

L151: "the number of overlapping SNPs as well as the average mismatch for unrelated pairs into account. We use the product of these two values as the "effective number of overlapping SNPs"

I find the term "effective number of overlapping SNPs" to be a bit misleading. As can be directly seen, the value is the *expected number of different alleles between this sample pair if they were unrelated*. So something like "expected mismatches" would be more fitting. I suggest explicitly describing this helpful interpretation.

Response: Thank you for this useful suggestion! We have updated the term to “expected number of mismatches” and explain at the first use that it is short for “expected number of different alleles between the sample pair if they were unrelated”.

A3) In Figure 2 and Figure 3, you show odd and non-matching numbers of simulation replicates. E.g., in Fig. 2 caption: "using simulated first-degree (n=118), second-degree (n=150), and third-degree pairs (n=144)".

It would be easier to describe and understand if a matching number is shown (and perhaps a larger number such as n=500, as n=100 can have substantial sampling uncertainty for intermediate fractions).

Response: Thank you for this suggestion! While we agree that larger numbers of tested pairs would add resolution, we do not think that the precision in individual estimates is crucial for the interpretation of our results. The simulated data were not generated for this article but for Aktürk et al (2024) and did not just involve pedigree simulation but also the simulation of ancient DNA sequence data and mapping. Consequently, a substantial amount of computational resources would be required to obtain a minor increase in resolution. As our interpretations are not based on individual face values but rather on broader patterns, we decided to not increase the number of simulated samples.

A3) L381-L383:

"READv2 is very similar in its approach to BREADR [33] and TKGWV2 [31], with each tool having its own unique feature. READv2 has the functionality to separate the different first-degree relationships, BREADR has a better quantification of uncertainty, and TKGWV2 works well with lower amounts of input data."

You summarize the relationship to other methods that use the same signal to detect relatives in two brief sentences. I think it would help to expand this discussion of the pairwise mismatch estimate ecosystem, and where you think READv2 will find its niche.

Response: We have a sentence on the place of READ in the biological relatedness ecosystem in our discussion section. Following the statement cited by the reviewer, we write: “The combination of increased efficiency and READ’s user-friendliness with a single input file qualify it as a QC step in data processing pipelines or as the first tool in an analysis of biological relatedness, which can be followed up with other tools to detect more fine-scale patterns or to verify results.”

A4) L473: "The normalization value was calculated as the median mismatch per subsample of the data."

This sentence in the section "Benchmarking" is the only time normalization and using the median are mentioned in the manuscript. Normalization is a key choice, and the online documentation discusses it extensively. It could help to move some of these explanations into the manuscript.

Response: We have added a discussion paragraph on the use and selection of an appropriate normalization value: “A READ-specific step before classification is the estimation of a normalization value expressing the expected P0 for a pair of unrelated individuals from the same population which serves as a baseline and helps to overcome potential biases arising from e.g. general population diversity or SNP ascertainment. Obtaining a good estimate is crucial and in default settings this value is estimated from the median of all pairwise comparisons in the dataset which usually works well for dataset sizes of 4 and higher if all individuals are from the same population [28]. There might be situations, however, where that approach could fail, especially for small sample sizes, e.g. for a trio (mother, father, child where only one out of three comparisons is unrelated). Therefore, READ offers the possibility to manually input a normalization value which could be estimated from a different dataset or only using the pairwise mismatch between the parents in a suspected trio. One could even consider multiple extractions from the same individual as technical replicates and then multiply their pairwise distance by 2 to obtain an expected value of unrelated individuals (as the expected normalized P0 for identical twins/duplicates is 0.5).”

B) Minor suggestions:

B1)

L108: "Using READv2 it was even feasible to analyze a full simulated dataset of 696 individuals and 241,860 pairs of individuals (see Methods and [38]) in less than 4 hours, but this required

237.5 GB of memory."

As a reader, one immediately compares these values to the "94 IIDs" and "5.64 GB" you mentioned above in the manuscript. My question was why the memory requirement is so much larger than just the factor of 7x (due to 7x more individuals). I guess that is the number of SNPs in the two datasets (1.2 million 1240k SNPs versus 8.7 million simulated WGS SNP), but it would be less of a "jump scare" if you make this point explicitly.

Response: Thank you for this observation! While re-running READv2 for the revisions, we noticed that READv2 was not run with the final default settings for this test.

READv2 now requires 65 GB for this particular dataset which is still more than 7 times the amount needed for the empirical dataset with 94 individuals. The memory needs seems to scale more than linearly with sample size (Figure 1C), so the 7-fold increased sample size will have a larger impact on the memory used. We decided to not investigate this further as sample sizes of ~700 for the detection of biological relatedness will likely remain rare and 65GB are usually available on cluster nodes.

B2)

L131: "READv2 seemed to perform slightly better for smaller compared to larger window sizes, but overall the genome-wide estimate worked best".

Isn't the genome-wide estimate effectively window size 1? So there is no "but".

Response: Thank you for the suggestion, but we consider the genome-wide estimate essentially an estimate for one single genome-wide window which would be the largest window possible. We have replaced the “but” with “and”.

B2)

L363 I had to look up the Latin abbreviation "viz.". Perhaps write "namely," as the Latin abbreviation seems to decline in use (even some native speakers do not know it anymore).

Response: We have removed “viz.” and replaced it with “i.e.”.

B3) In Table 1, only integers of the "Average number of effectively overlapping SNPs" are shown - which gets very coarse for low numbers (e.g., 5 in the last row). For low values, it would be useful to show at least two significant digits. Overall, that table could also easily be shown as a figure - where one would see the "scaling" with coverage more accurately and at one glance.

Response: We have now turned this table into a figure (Figure 3). This obviously reduces resolution for the reader even further. To retain some resolution, we added the average numbers to the plot in text form.

B4) L216: "Larger windows appear more suitable for this purpose (Figures S3 and S4), so we perform this analysis with 20Mb windows."

You do not give any actual reason why it is more useful. But the heuristic is simple—each meiosis in one of the two parents changes the IBD0/IBD1/IBD2 status, and there are only a few per chromosome. Thus, the stretches of the same status will be, on average, 50 cm long (and roughly 1cm = 1mb).

Response: Thank you for this excellent suggestion! We have now added “...as there will be very few IBD status changes along the chromosome with a segment length of 50cM on average (with 1cM ~ 1Mb in humans)”

B5) L222: "Cotterman coefficients k0 and k2". I believe that a citation would help. "Cotterman" is not a widely used nomenclature\

Response: Thank you, we added a citation.

B6) "This feature requires about 10,000 "effectively overlapping SNPs", which, assuming the popular 1240K SNP capture panel with 1.15 million autosomal SNPs and European Neolithic populations, would correspond to about 0.2X coverage per individual which is the case for a large proportion of all published human ancient genome-wide data [44]" As a substantial fraction of published aDNA is shotgun-sequenced, it would be very useful to also provide the corresponding coverage for that data type.

Response: We consider it difficult to quantify an expectation for WGS data as the exact number of polymorphic sites will depend on the population, variant calling and SNP ascertainment but WGS data certainly has the potential to provide SNP numbers larger than ~1.15 million. We have added: “As substantial amounts of generated data are also shotgun-sequenced, even lower coverages might allow for this type of analysis but the exact coverages and numbers of SNPs needed would depend on the SNP ascertainment and the population the study is focused on.”

B7) Table S1 (the comparison of different methods to detect relatedness in aDNA) is very informative. I would suggest considering moving it into the main, as it really helps to put READv2 into a broader context of methods.

Response: Thank you for this suggestion! We moved this table to the main text (Table 1 now).

C) Potential Updates to increase User-Friendliness

Here I provide some suggestions to increase the usability further. Those are entirely optional, and the authors should only go there if they believe it is a valuable use of their time.

C1) Packaging as proper Python package on pip

The authors could make their software (or effectively the .py file) a pip installable package. While, of course, not completely trivial, it is pretty straightforward (see https://packaging.python.org/en/latest/tutorials/packaging-projects/). Having READv2 as its pip package allows proper versioning and software dependencies on the necessary Python tools. Proper packaging would be an important step to include it in a widely used aDNA pipeline (e.g., Eager, which would benefit massively from a default step of detecting relatives) - further boosting the adoption.

Response: While the manuscript has been in review, we explored the possibility of preparing a READv2 bioconda recipe. This took longer than expected as the plinkio library was not available on bioconda. This is now fixed and we have updated the previous READv1 bioconda recipe to now provide version 2 (https://anaconda.org/bioconda/kinship-read). We expect that the bioconda recipe should, similar to the option of making it available through pip, allow easy integration into pipelines such as Eager.

C2) Using eigenstrat format as input

Eigenstrat (binary and non-binary) is widely used in aDNA (e.g., the Allen Ancient DNA resource is published in eigenstrat format), and most pseudo-haploid data is in this format. Of course, it is relatively straightforward to transform eigenstrat files into the READv2 Plink files (as the authors describe in their README). However, doing so creates an extra step requiring external software. There, it would be very user-friendly to provide the ability to analyze Eigenstrat files directly. Existing Python scripts can read both binary and non-binary eigenstat files, such as the one used by hapROH (see code at https://github.com/hringbauer/hapROH/blob/master/package/hapsburg/PackagesSupp ort/loadEigenstrat/loadEigenstrat.py).

Response: Thank you for this suggestion! The reviewer is correct that a lot of pseudohaploid data is published in eigenstrat format and that this could be added as a input file format. However, it is relatively easy to convert between the two formats. READv1 only had Plink TPED/TFAM and became widely used by the community. Furthermore, preparing the data in Plink formats are often also needed for other downstream analysis. Therefore, adding this option was not a priority for us during the limited time for the revision but we might consider adding the feature at a later point.

C3) READv2 has the nice feature of automatically classifying relatives (or refraining from doing so) using sensible coverage and statistical cutoffs. However, it would be very useful for the user to output the uncertainty of the final test statistic (the kinship coefficient theta) used for classification. As you have the bootstrap confidence intervals for PO - you could easily forward those CIs to theta and report those in the tabular output.

Response: Thank you for this suggestion! We added this as an extra column to the output file.

Reviewer #2: Alaçamlı and colleagues present an exciting update to the most widely used relatedness estimator in the field of archaeogenomics. This comes several years after the first release of READ (v1), and brings a welcomed update in speed, resource usage, and importantly, accuracy. As a frequent user of READv1, I am happy to see that the researchers remain interested in their own software, to the point that new features were developed, tested, and incorporated, and that the community's comments on how to possibly improve the software were listened to. Overall, the paper is well structured, in similar ways to other recent ancient DNA relatedness estimator papers. As the updated method is based on a peer-reviewed and well-established existing method, the authors dive right into the new features of READv2, and end up with a section where the method is applied to archaeogenomic data from a recent study.

Although I would happily recommend this paper to publication in Genome Biology, almost as is, I have a few small comments and observations that the authors could shed some more light into:

Results:

- Regarding the "Window size" section, perhaps a couple of extra sentences could be provided about the simulated data being used for these tests. Perhaps that will explain why only 1,878 SNPs overlap on average on pairs of individuals with 0.1X coverage data. From personal experience that coverage level should be able to provide way more overlapping SNPs than what is reported.

Response: After submission, we noticed that we had removed important information about the composition of the simulated data while trying to streamline the method section. The SNP data was downsampled to 200,000 SNPs which was the maximum number of variant sites that could be covered in an individual. That will explain the ~1900 overlapping SNPs for 0.1X coverage per individual (200000*0.1*0.1=2000). We have added this information to the methods section and apologize for the omission.

- I'm not completely convinced that using "effective number of overlapping SNPs" as a comparative threshold measure benefits the discussion and/or makes it clearer and more comparable. However, I am happy to change my mind! Mainly because minimum SNP (and/or coverage) thresholds are often used before using these methods to decide which samples should be used for relatedness estimation. Shall users then mostly use whole-genome coverages as the selecting threshold before running READv2? What about targeted-capture coverages? Also, see my comment below about "effective number of overlapping SNPs" not being comparable to all relatedness methods.

Response: We apologize if we came across as if we were suggesting to use the expected number of mismatches as a measurement for the inclusion of samples in an analysis or a dataset. We were not able to find any points in the text where we suggest this instead of the usual SNP number and/or coverage criteria. We merely suggest this measurement as a means for quantifying the amount of information available for the classification of a certain pair of individuals. Absolute numbers of SNPs or coverage are measurements per individual. We consider this number worth reporting in our output files in addition to the raw number of overlapping SNPs. The information content of overlapping SNPs will depend on their allele frequencies in the population which would not be considered when only reporting the absolute number. In general, we agree that no one dimensional measurement will be able to summarize all aspects and different situations ask for different types of information, but we believe that the expected number of mismatches is a useful way to express what data is available for comparing a certain pair of individuals.

- "Third-degree relationship": it would be useful for the reader to see false positive ratio numbers, for example when the 0.3X coverage is mentioned, just to have a more immediate understanding of the limits of this threshold.

Response: We have added the numbers for power and FPR to the respective paragraph and added an additional reference to the relevant figure (Figure 2).

- "Distinguishing between parent-offspring and siblings": On line 225 and after it is mentioned that from ~8000 effectively overlapping SNPs there is perfect separation between first degree classes. However, on line 262 and below (and Fig. 4) it is mentioned that 8000 effectively overlapping SNPs are still prone to misclassifications, while mentioning that the results are similar to what was achieved in the prior simulations. Are these differences due to different median/average mismatch rates? If effectively overlapping SNPs are supposed to normalise and make the results more comparable "across datasets with different SNP panels or population background diversities", how are the 8000 vs >14000 SNPs comparable to achieve similar successful classifications?

Response: After re-reading this section we have to agree with the reviewer that this point could require some additional information. We now elaborate more on the specific observations for each level of SNP numbers. There are numerous aspects in which different datasets can differ potentially causing such effects. While the normalization itself should handle aspects such as background diversity and SNP ascertainment, it does not account for features like background linkage disequilibrium which might explain why the approach seems to work better for the sub-Saharan YRI (lower LD) than the Asian CHS (higher background LD). Randomly drawing the simulated genotypes from the allele frequencies in the TSI population would have eliminated all background LD existing in that population which might explain why the distinction works best in this case as SNPs are more independent measurements. Therefore, it appears more important that we selected a conservative threshold of 10,000 expected mismatches.

- "Empirical data application: Rivollat et al. (2023)": In lines 332-335, did the authors compare the average/median mismatch rate of the original publication and their own? Could that be also a reason why the pair "GLN207A-GLN279" cross the threshold in one run but not another?

Response: Thank you for this suggestion! We checked the median P0 values from our READv2 run and the READv1 results provided by Rivollat et al. (2023). Our value is indeed slightly higher (0.2453 for the original study, 0.2488 in our re-analysis), and using our slightly higher value would have led to a first degree classification of the pair. We have added this observation to the text.

Discussion:

- "The introduction of "effectively overlapping SNPs" as a measurement of the amount of available data should also make studies and datasets more comparable and increase the possibility of generalizing from benchmarking results as previous studies mostly compared the raw number of overlapping SNPs or sequencing depth without taking the information content per SNP into account."

This can be true for comparing relatedness methods relying on pairwise mismatch rates, but won't be comparable to the methods described in Table S1 that use allele frequencies. That should be specifically mentioned.

Response: We have added the following statement to the discussion: “While the “expected number of mismatches” summarizes the allele frequency spectrum of the SNP panel in the population, numbers will not be directly comparable to approaches using the full population allele frequency information as additional input (Table 1).”

-Figure S1/S2: It's not crucial, but it would be interesting to also see the power and minimum data threshold of READv2 for "IdenticalTwins/SameIndividual" relationships.

Response: Thank you for the suggestion! The simulated dataset did not include sample duplicates. The simulated data were not generated for this article but for Aktürk et al (2024) and did not just involve pedigree simulation but also the simulation of ancient DNA sequence data and mapping. Consequently, a substantial amount of computational resources would be required to add this type of relatedness to the supplementary figure. In general, our expectation would be that the classification of "IdenticalTwins/SameIndividual" works better than first degree.

-Table S1: This is an extremely useful table for the community, but where does the 200,000 SNP panel of READv2 come from? I couldn't find a reference to it in the text, as it either mentions several million SNP datasets or the 1240K capture set. Also, for third degree the ~15,000 SNP number seems to not be the effectively overlapping SNPs, which makes sense when comparing with other methods, but in my view further makes things a bit more confusing.

Response: Following the suggestion from reviewer #1, we have now moved this table into the main manuscript. As mentioned before, we have also added to the methods section that our SNP panel was downsampled to 200,000 SNPs. Furthermore, we have added a footnote that the 15,000 SNPs in this table are an absolute but not an effective number.

Finally, the fact half of my comments/observations turned out to be regarding the way SNP thresholds are presented, attests to the quality and importance of the improvements of READv2 over its predecessor, and both as a reviewer and community member I am extremely happy to see it being published. Thanks for keeping on improving software in such a quickly moving field!

Response: We appreciate this positive assessment and want to thank the reviewer for the suggestions which helped clarify certain aspects of the study.

Reviewer #3: The study presents an advancement of the widely used tool READv1, designed for estimating kinships among ancient individuals using low-coverage data. This updated tool, named READv2, has been specifically tailored for Python 3 environments, introducing enhanced functionalities. It offers additional capabilities to identify third-degree relatives, as well as distinguishing between siblings and parent- offspring, both classified as first-degree relatives. As a user of READv1, I would like to see the paper ultimately published in this journal.

I only have a couple of questions. My small concern is the robustness of READv2 against ancient DNA damages, given their potential to influence pairwise mismatch rate estimations. Would the tool's performance improve if the sites in shotgun data were filtered for transversions?

This could be tested in the context of capture-sequenced data for 1240K sites or whole-genome shotgun sequenced data with an additional filter for minor allele frequency 1% based on global populations within the 1000 Genome Phase 3 dataset. Moreover, in instances where ancient DNA is not treated with UDG, are there any potential pitfalls in running READv2 or interpreting its results? Clarifying these aspects would greatly enhance the utility and reliability of the tool in diverse genomic contexts.

Response: We thank the reviewer for their kind words and the positive assessment of our manuscript.

Our simulated data included deamination damage. To make this important issue more visible to the readers, we have added a short discussion on the potential impact of post-mortem damage to the manuscript: “The preparation and filtering of input data is crucial for any ancient DNA analysis. For example, cytosine deamination (causing C>T and G>A changes) would increase the amount of mismatches in pairwise comparisons using both transition and transversion sites. While our simulated data included cytosine deamination, the extent was identical for all individuals which should elevate the number of mismatches for comparisons equally. In practice, we recommend either using data where such damages have been repaired enzymatically or restricting the analysis to transversion polymorphisms as unequal levels of deamination might bias pairwise mismatch statistics.” We are also adding recommendations to the README file on Github.

**2^nd^ round**

**Reviewer 2**

I am happy with the authors' responses to my previous comments, and would like to suggest this paper for acceptance.
